# Impact of corneal parameters, refractive error and age on density and morphology of the subbasal nerve plexus fibers in healthy adults

**DOI:** 10.1038/s41598-021-85597-5

**Published:** 2021-03-16

**Authors:** Anna M. Roszkowska, Adam Wylęgała, Romana Gargano, Rosaria Spinella, Leandro Inferrera, Bogusława Orzechowska-Wylęgała, Pasquale Aragona

**Affiliations:** 1 Ophthalmology Clinic, Department of Biomedical Sciences, University Hospital of Messina, Via Consolare Valeria, 98100 Messina, Italy; 2 Health Promotion and Obesity Management Unit, Pathophysiology Department, School of Medicine, Medical University of Silesia, Katowice, Poland; 3 Department of Economics, University of Messina, Messina, Italy; 4 Clinic of Otolaryngology, Head, Neck Surgery, Department of Pediatric Surgery, Medical University of Silesia, Katowice, Poland

**Keywords:** Eye manifestations, Nervous system, Visual system

## Abstract

The purpose of this study was to analyze corneal sub-basal nerve plexus (SBNP) density and morphology and their relationships with corneal parameters and refractive status. In this single center study, in vivo confocal microscopy (IVCM) was performed in 76 eyes of 38 healthy subjects aged 19–87 (mean age 34.987 ± 1.148). Nerve fiber analysis was performed using Confoscan 4 microscope with semi-automated software (Nidek Technologies, Italy) The nerve fiber length (NFL) µm/mm^2^, nerve fiber density (NFD) no./mm^2^, tortuosity coefficient (TC), and nerve beadings density (NBD) no./mm were considered. Relationship between SBNP parameters and corneal curvature, thickness, diameter, and refraction were analyzed. Additionally, the association with gender, laterality and age were determined. NFL was inversely correlated with age (r = − 0.528, p < 0.001), myopic refractive error (spherical value) (r = − 0.423, p < 0.001), and cylindrical power (r = − 0.340, p = 0.003). NFD was inversely correlated with age (r = − 0.420, p < 0.001) and myopic refractive error (r = − 0.341, p = 0.003). NBD showed a low inverse correlation with cylindrical power (r = − 0.287, p = 0.012) and a slight positive correlation with K (r = 0.230, p = 0.047). TC showed a significant negative correlation between age (r = − 0.500, p < 0.001) and myopic refractive error (r = − 0.351, p = 0.002). Additionally, there were strong positive correlations between NFL and NFD (r = 0.523, p < 0.001), NFL and TI (r = 0.603, p < 0.001), and NFD and TC (r = 0.758, p < 0.001). Multiple regression analysis revealed age to be the most significant factor affecting SBNP density (B = − 0.467, p = 0.013) and length (B = − 61.446, p < 0.001); myopic refractive error reduced both SBNP density (B = − 2.119, p = 0.011) and length (B = − 158.433, p = 0.016), while gender and laterality had no significant effects (p > 0.005). SBNP fiber length decreases with age, myopic refractive error and cylindrical power. SBNP fiber density reduces with age and myopic refractive error. Corneal nerve parameters are not influenced by gender or laterality.

## Introduction

The cornea is the most densely innervated tissue in the human body. Corneal sensory innervation derives from the ophthalmic branch of the trigeminal nerve and reaches the corneoscleral limbus with the anterior ciliary nerves. The nerves enter the mid-corneal stroma from the corneal limbus. Below the basal epithelium, the corneal nerves form the sub-basal plexus (SBNP) that runs parallel to the corneal surface below the epithelial layer. SBNP fibers in the healthy human cornea show a whorled pattern pointed at the lower nasal quadrant^1–3^. In vivo confocal microscopy (IVCM) is a method that allows the depiction of the cornea on a histological scale and is valuable in assessing corneal properties. Using IVCM, it is possible to analyze all corneal layers, to measure the corneal thickness, endothelial cell density, depth of lesions, and reflectance^2–7^. With IVCM the SBNP can be examined and the corneal confocal microscopy (CCM) parameters such as nerve fiber density (NFD), nerve fiber length (NFL), nerve beading density (NBD), and tortuosity coefficient (TC) can be measured^2,3,7–10^.

Nerve fibres length (NFL) is defined as the total length of all nerve fibres within the area and represented in mm/mm^2^. Nerve fibres density (NFD) is the total number of major nerves per millimeter squared and represented in no./mm^2^^11–14^. Some authors considered nerve fibers density (CNFD) which represents the fibers length density per area using the unit mm/mm^2^ that differs from the previously defined NFD^4,5,15,16^. The NFL is considered as a more stable parameter and it is diminished in different corneal conditions^2,3,7–10^. It was determined as the optimal and the most reliable parameter for the detection of diabetic sensorimotor polyneuropathy^17–19^.

Nerve beading density (NBD) expressed in no./mm represents the total number of nerve beadings divided by the total length of nerve trunks in millimeter. Beadings are efferent to the axons and sensory terminals, and consist of accumulations of mitochondria and glycogen^8–10,20^.

Nerve fibers tortuosity is expressed by the unitless measure representing the degree of twistedness of fibers and calculated according to the Kallinikos’ tortuosity coefficient (TC), and its higher number indicates the more tortuous nerves. Tortuosity increment, sign of nerve damage, indicates the regeneration occurring in damaged nerves with active fibers growth^21,22^. Both higher tortuosity and beading density are considered signs of higher metabolic activity of the SBNP responding to epithelial changes^21–23^.

Different studies investigated SBNP parameters in healthy cornea and considerable differences in normal values of the SBNP parameters reported by investigators could be attributed to variations in study design, different devices used to perform IVCM, and different methods of nerve assessment (manual, semi-automated, or automated)^2,5–9,20,22,24,25^.

Previous studies reported NFD, NFL, NBD, and TC in health and disease, but there have been no reports investigating whether corneal parameters might influence SBNP density and morphology^2,7,8,10,22,23^. Here, we aimed to investigate whether SBNP parameters in the healthy cornea, are related to the corneal parameters such as central corneal thickness (CCT), corneal curvature (K), corneal diameter (CD), and refractive status. Furthermore, we analyzed the impact of demographics such as gender and age on changes in SBNP parameters.

## Subjects and methods

The study was performed on 76 eyes of 38 healthy subjects (22 F and 16 M) aged from 19 to 87 years (mean 34.987 ± 1.484). The patients were recruited from candidates for laser refractive surgery and from the general ophthalmology ambulatory where they presented for the periodic ocular examination. The refractive errors were measured with both subjective evaluation and autorefractive readings (Topcon, Japan) and the values were determined by assessment of the best corrected visual acuity. Individuals with any systemic diseases were excluded and only subjects with no ocular disease or history of ocular surgery were enrolled. The patients were not contact lenses wearers and had a best-corrected visual acuity of 20/20. The informed consent was obtained from all participants. The study was approved by the Ethical Committee of the University Hospital of Messina and adhered to the tenets of the Helsinki Declaration.

### Corneal parameters measurement

Slit lamp examination was performed prior to the instrumental examinations. Corneal curvature was measured with corneal topography (Antares, CSO^®^, Scandicci, Italy) and Average K readings were considered. The corneal horizontal diameter was determined on videokeratography images. Three measurements of the central corneal thickness (CCT) were performed with ultrasound pachymeter (Optikon 2000^®^, Italy), by the same observer after topical instillation of unpreserved 0.4% oxybuprocaine (Novesina; Novartis Farma, Origgio, Italy) and the mean value was calculated and expressed in microns. IVCM was performed using the slit scanning confocal microscope (Confoscan 4, Nidek Technologies^®^, Vigonza, Italy) after topical instillation of unpreserved 0.4% oxybuprocaine (Novesina; Novartis Farma, Origgio, Italy). The examination was performed using the 40 × contact objective, provided with Z-Ring probe to allow precise positioning of the lens over the central corneal area. An ophthalmic gel 0.2% carbomer (Viscotears; Novartis Farma, Italy) was used to improve the adhesion of the objective to the cornea. Complete scanning of the cornea was carried out three times for each eye.

### Sub-basal Nerve Plexus analysis

The nerve fiber analysis was performed on images obtained from the subepithelial layers, where the sub-basal nerve plexus is present. Only well focused, clear images (460 × 345 mm) were considered with three images per each corneal scan for a total of nine photographs for single eye.

**Figure 1:**
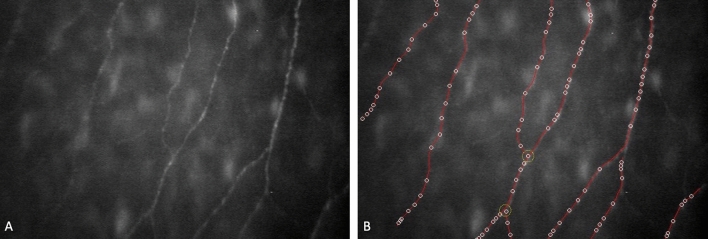
The source image of the SBNP fibers (**A**) and the segmentation sample (**B**). The software traces nerve fibers and detects the beadings and automatically provides CCM values.

The selected images were evaluated by one experienced operator using the semi-automated nerve analysis software program (Nidek Technologies, Italy). The software traces automatically nerve fibers, evidences beadings, provides CCM parameters and calculates tortuosity coefficient (Fig. 1). We define NFL as the total length of all nerve fibres within the area and expressed in mm/mm^2^, NFD as the total number of major nerves per millimeter squared represented in no./mm^2^ and NBD as the total number of nerve beadings divided by the total length of nerve fibres in millimeter reported in no./mm. The operator was enabled to trace the fibers undetected by the program that were successively incorporated into analysis. In this study the values obtained for NFL, NFD, NBD and the TC were considered and the correlation with corneal parameters, age and refractive status was investigated.

### Statistical analysis

Preliminary, the data were assessed for normality using the Shapiro–Wilk normality test. In order to determine any significant differences between gender and laterality for all corneal parameters Student *t* test for independent sample was applied. Bravais–Pearson correlation test was performed to measure the type and intensity of the linear relationship between the variables. The multiple linear regression model was applied to study the effects of independent variables (corneal parameters and age) on the dependent variable (corneal nerve parameters) as a function of a given theoretical model, as well as to identify a linear combination of independent variables to optimally predict the value assumed by the dependent variable. The multicollinearity absence between variables was verified by tolerance and variance inflation factor (VIF). The assumptions of a linear regression model were tested on residual plots.

Stata 15.1 (StataCorp, College Station, TX, USA) was used to perform statistical analyses.

### Ethics approval

This study was approved by the Ethical Committee of the University Hospital of Messina and adhered to the tenets of the Helsinki Declaration.

### Consent for publication

All authors consented to publication of this manuscript.

## Results

**Figure 2:**
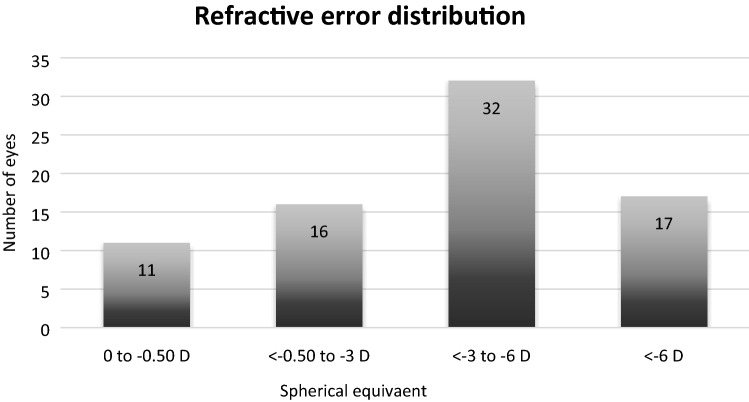
Distribution of the myopic refractive error in the examined eyes.

Table 1 reports the descriptive statistics for all quantitative variables. Most of the participants presented myopic refractive error that ranged from − 0.5 to − 11.25 diopters, with an average sphere value of − 4.125 ± 0.321 D, and an average astigmatism of − 0.694 ± 0.092 D (range − 0.5 to − 3.5 D). The distribution of refractive error is represented in the Fig. 2. The average K was 43.932 ± 0.159 D, while mean corneal diameter equaled 12.050 ± 0.044 mm. The mean central corneal thickness across all individuals was 563.027 ± 3.716 µm.

**Table 1:** Statistical summary of the collected eye data*.

	Min	Max	Mean	S error	S deviation
NFL (µm/mm^2^)	1385.540	12,082.350	8515.546	206.899	1791.799
NFD (no./mm^2^)	21.940	124.310	68.076	2.327	20.291
NBD (no./mm)	15.230	57.660	36.958	1.104	9.564
TC	2.520	38.380	21.536	0.833	7.218
Age (years)	19.000	87.000	34.987	1.484	12.937
K (D)	40.990	46.850	43.932	0.159	1.387
CD (mm)	11.040	12.830	12.051	0.044	0.377
CCT (mm)	482.000	634.000	563.027	3.716	32.181
Sph (D)	− 11.250	0.000	− 4.125	0.321	2.795
Cyl (D)	− 3.500	0.000	− 0.694	0.092	0.806

*NFL nerve fiber length (µm/mm^2^), *NFD *nerve fiber density (no./mm^2^), *NBD *nerve beadings density (no./mm), *TC *tortuosity coefficient, *K *corneal curvature (*D *diopters), *CD *corneal diameter (mm), *CCT *corneal thickness (µm), *Sph *sphere (*D *diopters), *Cyl *cylinder (*D *diopters).

### Laterality

**Figure 3:**
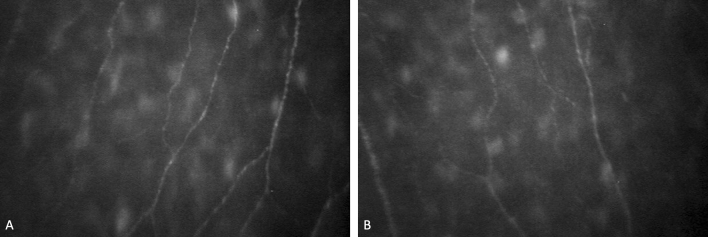
The example of the SBNP fibers from the left eye (**A**) and the right eye (**B**) of the same subject with low myopia.

No corneal parameters were significantly different between right and left eyes (Table 2): average K (p = 0.544), corneal diameter (p = 0.521), CCT (p = 0.634), spherical power (p = 0.737), cylindrical power (p = 0.608), NFL (p = 0.495), NFD (p = 0.445), NBD (p = 0.583), and TC (p = 0.901) (Fig. 3).

**Table 2:** Descriptive statistics for eyes’ laterality (*L *left; *R *right) and test t p-value*.

	Laterality	Mean	S deviation	S error	p-value
NFL (µm/mm^2^)	L	8371.242	1922.583	316.071	0.495
	R	8656.053	1668.401	270.650	
NFD (no./mm^2^)	L	69.916	19.094	3.139	0.445
	R	66.329	21.465	3.437	
NBD (no./mm)	L	37.595	10.539	1.757	0.583
	R	36.369	8.665	1.387	
TC	L	21.642	7.161	1.177	0.901
	R	21.433	7.368	1.195	
K (D)	L	44.032	1.362	0.224	0.544
	R	43.837	1.421	0.228	
CD (mm)	L	12.080	0.341	0.057	0.521
	R	12.023	0.411	0.067	
CCT (mm)	L	561.167	33.334	5.556	0.634
	R	564.744	31.416	5.031	
Sph (D)	L	− 4.014	2.703	0.444	0.737
	R	− 4.231	2.911	0.466	
Cyl (D)	L	− 0.743	0.867	0.143	0.608
	R	− 0.647	0.752	0.120	

*NFL nerve fiber length (µm/mm^2^), *NFD *nerve fiber density (no./mm^2^), *NBD *nerve beadings density (no./mm), *TC *tortuosity coefficient, *K *corneal curvature (*D *diopters), *CD *corneal diameter (mm), *CCT *corneal thickness (µm), *Sph *sphere (*D *diopters), *Cyl *cylinder (*D *diopters).

### Corneal parameters and refraction between genders

Corneal nerves parameters were not significantly different between genders: NFL (p = 0.211), NFD (p = 0.512), NBD (p = 0.933), TC (p = 0.284). However, significant differences were observed in average K (p = 0.009), corneal diameter (p = 0.035) and spherical power (p < 0.001) (Table 3). Specifically, the mean K value was 43.452 ± 0.223 D and 44.281 ± 0.208 D, the mean diameter was 12.162 ± 0.054 mm and 11.975 ± 0.062 mm, and the mean spherical power was − 2.797 ± 0.357 D and − 5.091 ± 0.437 D in men and women, respectively.

**Table 3:** Descriptive statistics for gender (16 M = male; 22 F = female) and test *t* p-value*.

	Gender	Mean	S deviation	S error	p-value**
NFL (µm/mm^2^)	M	8205.988	2126.351	381.904	0.211
	F	8733.644	1500.973	226.280	
NFD (no./mm^2^)	M	66.272	23.271	4.114	0.512
	F	69.387	17.987	2.712	
NBD (no./mm)	M	37.067	10.105	1.786	0.933
	F	36.876	9.261	1.412	
TC	M	20.494	8.489	1.501	0.284
	F	22.312	6.096	0.930	
Age (years)	M	36.031	16.370	2.894	0.552
	F	34.227	9.866	1.487	
K (D)	M	43.452	1.263	0.223	**0.009**
	F	44.281	1.382	0.208	
CD (mm)	M	12.162	0.295	0.054	**0.035**
	F	11.975	0.410	0.062	
CCT(mm)	M	565.161	32.464	5.831	0.633
	F	561.523	32.270	4.865	
Sph (D)	M	− 2.797	2.020	0.357	**< 0.001**
	F	− 5.091	2.901	0.437	
Cyl (D)	M	− 0.664	0.834	0.147	0.784
	F	− 0.716	0.793	0.120	

*M male, *F *female, *NFL *nerve fiber length (µm/mm^2^), *NFD *nerve fiber density (no./mm^2^), *NBD *nerve beadings density (no./mm), *TC *tortuosity coefficient, *K *corneal curvature (*D *diopters), *CD *corneal diameter (mm), *CCT *corneal thickness (µm), *Sph *sphere (*D *diopters), *Cyl *cylinder (*D *diopters).

**Bold values denote statistical significance at the p value < 0.05.

### SBNP parameters

Table 4 reports the correlations between all variables considered in this study, and Table 5 shows the results of the four multiple regression models for the dependent variables NFL, NFD, NBD and TC respectively.

**Table 4:** Pearson correlation matrix.

		NFL (µm/mm^2^)	NFD (no./mm^2^)	NBD (no./mm)	TC	Age (years)	K (D)	CD (mm)	CCT (mm)	Sph (D)	Cyl (D)
NFL (µm/mm^2^)	R	1	0.523	0.190	0.603	− 0.528	− 0.053	− 0.024	− 0.058	− 0.423	− 0.340
	p-value*		**0.000**	0.105	**0.000**	**0.000**	0.655	0.836	0.625	**0.000**	**0.003**
NFD (no./mm^2^)	R	0.523	1	− 0.093	0.758	− 0.420	− 0.121	0.187	− 0.190	− 0.341	− 0.165
	p-value*	**0.000**		0.426	**0.000**	**0.000**	0.298	0.110	0.103	**0.003**	0.155
NBD (no./mm)	R	0.190	− 0.093	1	− 0.051	− 0.107	0.230	0.008	− 0.013	− 0.151	− 0.287
	p-value*	0.105	0.426		0.666	0.362	**0.047**	0.944	0.909	0.197	**0.012**
TC	R	0.603	0.758	− 0.051	1	− 0.500	− 0.047	0.125	− 0.132	− 0.351	− 0.155
	p-value*	**0.000**	**0.000**	0.666		**0.000**	0.691	0.293	0.262	**0.002**	0.183
Age (years)	R	− 0.528	− 0.420	− 0.107	− 0.500	1	− 0.059	− 0.321	0.174	0.275	0.076
	p-value*	**0.000**	**0.000**	0.362	**0.000**		0.616	**0.005**	0.136	**0.016**	0.511
K (D)	R	− 0.053	− 0.121	0.230	− 0.047	− 0.059	1	− 0.463	0.102	− 0.067	0.208
	p-value*	0.655	0.298	**0.047**	0.691	0.616		**0.000**	0.386	0.566	0.072
CD	R	− 0.024	0.187	0.008	0.125	− 0.321	− 0.463	1	− 0.035	0.123	− 0.150
	p-value*	0.836	0.110	0.944	0.293	0.005	0.000		0.770	0.296	0.201
CCT (mm)	R	− 0.058	− 0.190	− 0.013	− 0.132	0.174	0.102	− 0.035	1	− 0.142	0.193
	p-value*	0.625	0.103	0.909	0.262	0.136	0.386	0.770		0.225	0.096
Sph (D)	R	− 0.423	− 0.341	− 0.151	− 0.351	0.275	− 0.067	0.123	− 0.142	1	0.077
	p-value*	**0.000**	**0.003**	0.197	**0.002**	**0.016**	0.566	0.296	0.225		0.510
Cyl (D)	R	− 0.340	− 0.165	− 0.287	− 0.155	0.076	0.208	− 0.150	0.193	0.077	1
	p-value*	**0.003**	0.155	**0.012**	0.183	0.511	0.072	0.201	0.096	0.510	

*NFL nerve fiber length (µm/mm^2^), *NFD *nerve fiber density (no./mm^2^), *NBD *nerve beadings density (no./mm), *TC *tortuosity coefficient, *K *corneal curvature (*D *diopters), *CD *corneal diameter (mm), *CCT *corneal thickness (µm), *Sph *sphere (*D *diopters), *Cyl *cylinder (*D *diopters).

**Bold values denote statistical significance at the p value < 0.05.

**Table 5:** Multiple regression model—dependent variable NFL, NFD, NBD and TC*.

SNP parameter	Variables	B non standardized	SD	B standardized	P value*	Tolerance	VIF
NFL (µm/mm^2^)	Constant	28,349.19	11,070.01		**0.013**		
	Age (years)	− 61.446	14.505	− 0.447	**< 0.001**	0.753	1.328
	K (D)	− 176.681	138.564	− 0.138	0.207	0.718	1.392
	CD (mm)	− 1030.58	539.281	− 0.215	0.060	0.660	1.516
	CCT (mm)	2.466	5.415	0.044	0.650	0.885	1.129
	Sph (D)	− 158.433	63.866	− 0.249	**0.016**	0.832	1.201
	Cyl (D)	− 656.134	212.06	− 0.297	**0.003**	0.908	1.101
NFD (no./mm^2^)	Constant	145.537	140.073		**0.042**		
	Age (years)	− 0.467	0.184	− 0.298	**0.013**	0.753	1.328
	K (D)	− 1.407	1.753	− 0.096	0.425	0.718	1.392
	CD (mm)	3.959	6.824	0.073	0.564	0.660	1.516
	CCT (mm)	− 0.101	0.069	− 0.159	0.146	0.885	1.129
	Sph (D)	− 2.119	0.808	− 0.292	**0.011**	0.832	1.201
	Cyl (D)	− 1.464	2.683	− 0.058	0.587	0.908	1.101
NBD (no./mm)	Constant	− 117.051	69.774		**0.048**		
	Age (years)	0.012	0.091	0.009	0.845	0.753	1.328
	K (D)	2.423	0.873	0.354	**0.007**	0.718	1.392
	CD (mm)	3.548	3.399	0.139	0.300	0.660	1.516
	CCT(mm)	0.01	0.034	0.001	0.902	0.885	1.129
	Sph (D)	− 0.407	0.403	− 0.12	0.315	0.832	1.201
	Cyl (D)	− 3.911	1.337	− 0.332	**0.005**	0.908	1.101
TC	(Constant)	57.13	48.439		0.242		
	Age	− 0.237	0.063	− 0.427	**< 0.001**	0.753	1.328
	K (D)	− 0.375	0.606	− 0.073	0.538	0.718	1.392
	CD (mm)	− 0.393	2.36	− 0.02	0.868	0.660	1.516
	CCT(mm)	− 0.016	0.024	− 0.072	0.498	0.885	1.129
	Sph (D)	− 0.612	0.279	− 0.239	**0.032**	0.832	1.201
	Cyl (D)	− 0.673	0.928	− 0.076	0.471	0.908	1.101

*NFL nerve fiber length (µm/mm^2^), *NFD *nerve fiber density (no./mm^2^), *NBD *nerve beadings density (no./mm), *TC *tortuosity coefficient, *K *corneal curvature (*D *diopter), *CD *corneal diameter (mm), *CCT *corneal thickness (µm), *Sph *sphere (*D *diopter), *Cyl *cylinder (*D *diopter).

**Bold values denote statistical significance at the p-value < 0.05.

#### Nerve fibers length

**Figure 4:**
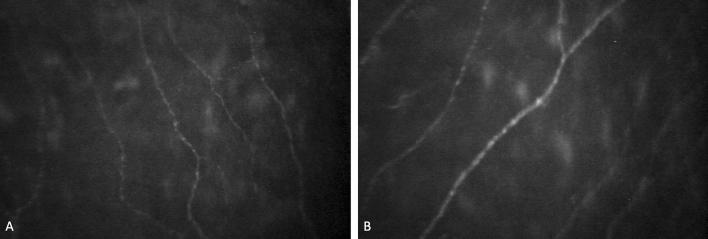
The SBNP fibers picture of the eye with emmetropia (**A**) and with a high myopia (**B**).

Mean NFL was 8515.546 ± 206.899 µm/mm^2^ and significantly inversely correlated with age (r = − 0.528, p < 0.001), spherical power (r = − 0.423, p < 0.001), and cylindrical power (r = − 0.340, p = 0.003). No statistical correlations were found with average K (r = − 0.053, p = 0.655), corneal diameter (r = − 0.024, p = 0.836) and CCT (− 0.058, p = 0.625). Mean NFL was strong positively correlated with mean NFD (r = 0.523, p < 0.001) and mean tortuosity (r = 0.603, p < 0.001). The multiple linear regression model (Table 5) showed that age (< 0.001), spherical power (p = 0.016), and cylindrical power (p = 0.003) negatively influenced the mean NFL. Standardized coefficients showed that age is the most influential variable (beta standardized = B = − 0.447. p < 0.001) followed by cylindrical power (B = − 0.297, p = 0.003) and spherical power (B = − 0.249, p = 0.016). The results show that a decrease of 61.446 µm/mm^2^ can be expected with an age increase of 1 year, assuming that all other variables in the models are held constant. Similarly, NFL decreases on average by − 158.433 µm/mm^2^ for each increase in the spherical power and decreases by − 656.134 µm/mm^2^ when the cylindrical power increases by one unit, when all other variables are constant (Fig. 4).

The information in the table allows us to check multicollinearity absence in the model; the tolerance was > 0.1 and VIF < 10 for all variables.

#### Nerve fibers density

The mean NFD was 68.076 ± 2.327 no./mm^2^ and was significantly negatively correlated with age (r = − 0.420, p < 0.001) and spherical power (r = − 0.341, p = 0.003), but not with average K (r = − 0.121, p = 0.298), corneal diameter (r = 0.187, p = 0.110), CCT (− 0.190, p = 0.103), or cylindrical power (r = − 0.165, p = 0.0155). Mean NFD was strong positively correlated with mean NFL (r = 0.523, p < 0.001) and mean tortuosity (r = 0.758, p < 0.001).

NFD was statistically dependent only on age (p = 0.013) and spherical power (p = 0.011). The two corneal parameters had a similar negative influence; the standardized coefficients were B = − 0.298 (age) and B = − 0.292 (spherical power). Results showed a mean decrease of NFD of 0.467 mm^2^ could be expected with an increase of one year in age, assuming that all other variables in the models are held constant. A mean NFD reduction of − 2.119 fibers/mm^2^ can be expected for every unit increase of spherical power, assuming all other variables are constant (Table 5). There was not multicollinearity between variables (tolerance > 0.1 and VIF < 10 for all variables).

#### Nerve beadings density

The mean NBD was 36.958 ± 1.104/mm and had a low correlation with cylindrical power (r = − 0.287, p = 0.012) and K (0.230, p = 0.047). There was no correlation between NBD and all variables considered here: age (r = − 0.107, p = 0.362), corneal diameter (r = 0.008, p = 0.944), CCT (r = − 0.013, p = 0.909), spherical power (r = − 0.151, p = 0.197). Nerves beadings density was not correlated with any corneal nerve parameter: NFL (r = 0.190, p = 0.105), NFD (r = − 0.093, r = 0.426), mean TC (r = − 0.051, p = 0.666).

NBD was statistically dependent by average K (p = 0.007) and cylindrical power (p = 0.005) with more impact of average K (standardized coefficient B = 0.354, p = 0.007) respect to cylindrical power (B = − 0.332, p = 0.005). We could expect mean increase NBD of 2.432 beadings/mm when K increases one unit, assuming that all other variables in the model are held constant and a decrease of − 3.991 beadings/mm in NBD mean for every unit increase of cylindrical power, always assuming other variables as constants (Table 5).

#### Nerves tortuosity coefficient

The mean nerve tortuosity index was 21.536 ± 0.833. There was a significant negative correlation between tortuosity and age (r = − 0.500, p < 0.001) and spherical value (r = − 0.351, p = 0.002). There was no significant correlation with average K (r = − 0.047, p = 0.691), corneal diameter (r = 0.125, p = 0.293), CCT (− 0.132, p = 0.262) and cylindrical power (− 0.155, p = 0.183). Additionally, nerve tortuosity was strongly positively correlated with mean NFL (r = 0.603, p < 0.001) and mean NFD (r = 0.758, p < 0.001). Table 5 shows that age (p < 0.001) and spherical value (p = 0.032) contributed significantly to mean tortuosity and that age had the greatest impact (standardized coefficient B = − 0.427 and B = − 0.239, respectively). Moreover, it can be expected that an increase by one year of age will decreases tortuosity in mean of 0.237 and that a decrease in one unit of spherical power will increases tortuosity in mean of 0.673, when all other variables are constant.

## Discussion

IVCM, is a minimally invasive method that does not require complex infrastructure and allows the visualization of corneal nerves in clinic. Since the confocal examination became possible in the clinic, several reports on corneal nerve fibres structure in health and disease were published showing alterations in both systemic and ocular conditions and after corneal refractive surgery^2,6–8,10,21–23,25^. Particularly the IVCM proved to be effective in monitoring small fibres diabetic neuropathy with conventional CCM morphometry and novel imaging and analysis techniques^10,21,26^.

Different studies were performed to investigate normal SBNP parameters with different confocal microscopy devices.

As to the NFL, the scanning confocal microscope (SSCM) studies report values that range from 6.1 ± 1.2 to 13.5 ± 0.3 mm/mm^2^ and those obtained with laser scanning confocal microscope (LSCM) showed the range from 16.1 to 26.4 mm/mm^2^.

The normal values of NFD obtained with the SSCM ranges from 26.5 ± 7.5 to 45.6 ± 4.5 fibers/mm^2^. The data related to the normal beadings frequency range from 90 ± 18 to 222 ± 43 beads/mm^2^ and tortuosity coefficient from 1.09 ± 0.54 to 2.2 ± 0.9^5–8,11,21,22,27^.

These wide ranges of normality result from the use of different devices and adoption of different modalities of analysis. The devices used in these studies were the tandem scanning, scanning slit and laser scanning confocal microscopes^28^. Tandem scanning is no longer used due to its poor resolution, which provides inferior values. The SCCM and the LSCM are currently still in use, and both provide a high quality of nerve imaging^8^. However, to perform a comparative analysis of nerve characteristics in different groups, the same device must be used to avoid erroneous interpretation^1–3^. Nevertheless numerous studies on normal corneal SBNP nerves were conducted to the best of our knowledge there are no reports on the relation between SBNP morphology and density and corneal parameters and refraction in healthy eyes. In this study we performed analysis of the SBNP nerve parameters such as NFD, NFL, NBD, and TC in healthy subjects, aiming to investigate their relationship with corneal parameters and refractive status of the eye. Additionally, the laterality and correlation with gender and age were assessed.

In the present study we found negative correlations between NFL, NFD and age. Some studies have shown no correlation between age and nerve density in healthy participants^3,29^. However, these studies were cross-sectional in their character. Deghani et al., who reported NFL in mm/mm^2^, in their 3 year-long study, showed an effect of age on NFL with a linear decrease of 0.05 mm/mm^2^ per year^12^. And Gruptcheva et al. showed a significant difference in nerves fibers density between two groups with mean ages of 25 and 70^30^. Tavakoli et al., in the study on 343 healthy participants, showed a decrease in NFD of − 0.164 no./mm^2^ per year for man and − 0.161 no./mm^2^ per year for woman and decrease in NFL of − 0.045 mm/mm^2^ and − 0.060 mm/mm^2^ per year respectively. As in our study, they found increased tortuosity in aged corneas^31^.

Our results show that a decrease of 61.446 µm/mm^2^ can be expected with an age increase of 1 year, assuming that all other variables in the models are held constant. Similarly, NFL decreases on average by − 158.433 µm/mm^2^ for each increase in the spherical power and decreases by − 656.134 µm/mm^2^ when the cylindrical power increases by one unit, when all other variables are constant.

Our results show a greater decrement of NFD (0.467 mm^2^/year) confirming that aging has a significant effect on corneal nerves fibres leading to the reduction of both density and length. Batawi et al. showed a negative correlation of NFD, NFL, and beadings with age with r = − 0.471, − 0.461, and − 0.310, respectively, while the TC was not affected^20^. Similarly to our study, the authors found that NFL correlated with other parameters (NFD, NBD, TI) with r > 0.52. The correlations between the assessed parameters can be explained by the nature of the parameters. For instance, an increase in NFL and tortuosity will naturally lead to an increase in NFD; such correlations can be seen as strength of the analysis.

Although we cannot fully explain the correlations between NBD, cylindrical power and total corneal power, we hypothesize that the correlation between K and cylindrical power is an effect of the different appearance of nerves in cylindrically shaped corneas. Alternatively, this correlation is subtle, and although statistically significant, it does not represent a real difference. We showed that an increase of 1 D in K leads to a 2.432 no./mm increase in NBD, and a 1-D increase in cylindrical power leads to a decrease of − 3.991 no./mm. Surprisingly, neither CCT nor corneal diameter showed any effect on the studied parameters. The lack of correlation may be explained by the nature of the SBNP and its location in particular part of the cornea.

Harrison et al. using the same confocal system but different software, showed a negative correlation between myopia and nerve density but they did not perform regression analysis between other corneal parameters^32^. In this study we measured spherical power and not axial length, but as Harrison et al. showed, the spherical power is a much better predictor of SBNP density.

Finally, we found, accordingly to other reports, that there were no differences between the left and right eye nor between genders in the IVCM parameters^2,3,7–13,20,21,30,31^.

So, it can be concluded that the multiple regression analysis confirmed that the age is the most significant factor affecting SBNP nerves density, length and tortuosity. Myopic refractive error (spherical power) reduces both the SBNP density and length and NFL, NFD, NBD and TC are not affected by gender or eye laterality. We believe that further studies including eyes with hyperopia and small corneal diameter are necessary to evidence furthermore the relationship between the refractive status of the eye, corneal morphology and SBNP parameters.

## Data Availability

Data is available from the corresponding author upon request.
